# Supplementary figures and images for: Ciliated cell domains with locally coordinated ciliary motion generate a mosaic of microflows in the brain’s lateral ventricles
Source: bioRxiv. 2025 Feb 20:2025.02.19.638730. Preprint. [Version 1] doi: 10.1101/2025.02.19.638730 (PMC11870595; doi:10.1101/2025.02.19.638730)

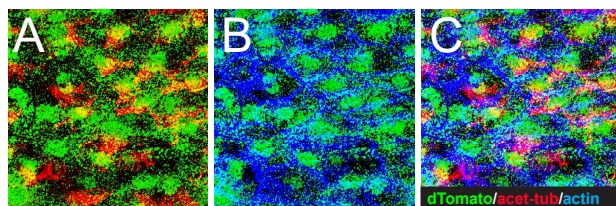

Supplement: Supplement 1 — Supplementary Figure1/SF1 Expression of nNOS in the nNOS-CreER/Ai9 mice. dTomato expression, driven by nNOS promoter, is observed in the ciliated cells, with the sample counterstained with antibodies to anti-dTomato, acetylated α-tubulin (red) and actin (blue). Scale bar is 10 μm for (A-C). After inducing nNOS-driven recombination with tamoxifen, dTomato signal is observed in the ependymal cells of the lateral ventricular wall. Co-staining with antibodies to dTomato, acetylated alpha-tubulin (a ciliary marker) and phalloidin (to highlight the apical actin cytoskeleton) revealed dTomato signal on the apical cell surface of the ciliated cells, concentrated in the tufts of cilia, implying nNOS expression in the multiciliated cells of the ventricles [file media-1.pdf]

WT

nNOS KO

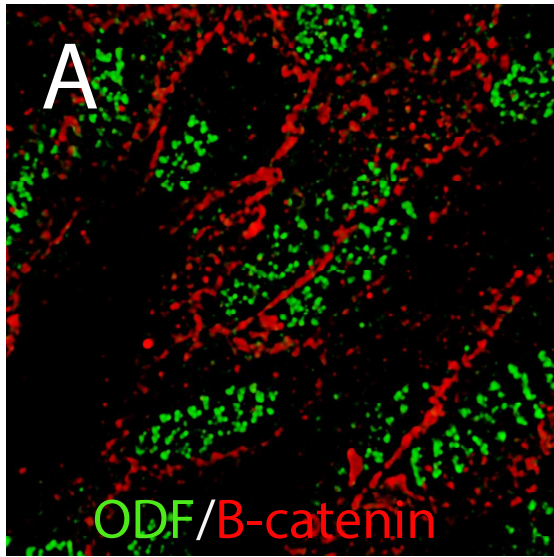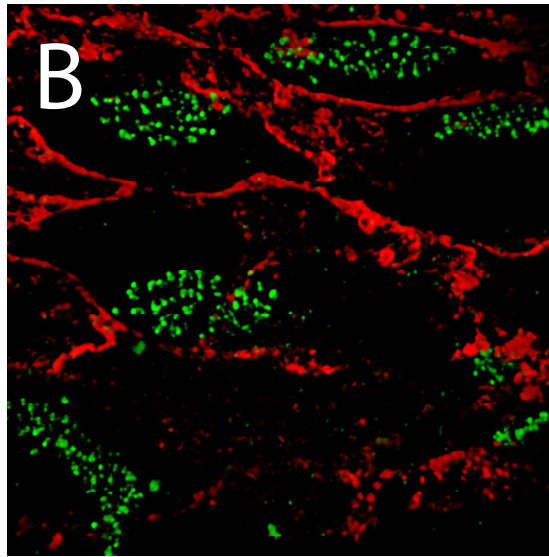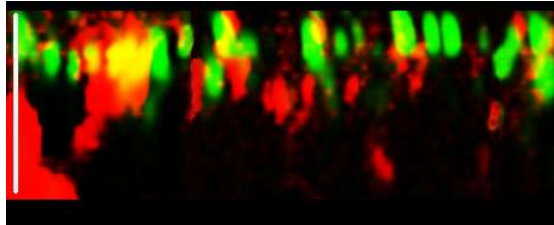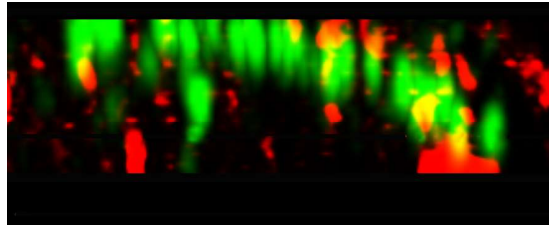

Supplement: Supplement 2 — Supplementary Figure 2 /SF-2 nNOS deletion does not cause defects in the basal bodies docking [file media-2.pdf]
