# Supplementary material for: Ciliated cell domains with locally coordinated ciliary motion generate a mosaic of microflows in the brain’s lateral ventricles
Source: bioRxiv. 2025 Feb 20:2025.02.19.638730. Preprint. [Version 1] doi: 10.1101/2025.02.19.638730 (PMC11870595; doi:10.1101/2025.02.19.638730)
Supplement: Supplement 8 [file media-8.pdf]

Supplemental data

Supplementary Figures:

SF1 **Expression of nNOS in the nNOS-CreER/Ai9 mice**

SF-2 **nNOS deletion does not cause defects in the basal bodies docking**

Supplementary movies:

S1-S3, represented as frames in Figure 4, cilia motion in wild-type

S4 Cilia motion in the ependyma of WT

S5-S6 represented **as frames in Figure 4**, cilia motion in nNOS KO

S7 Cilia motion in the ependyma of nNOS KO

S8 Cilia motion in **WT, high magnification**

S9 Cilia motion in **nNOS KO, high magnification**
